# Exogenous Melatonin Alleviates Skeletal Muscle Wasting by Regulating Hypothalamic Neuropeptides Expression in Endotoxemia Rats

**DOI:** 10.1007/s11064-021-03489-6

**Published:** 2022-01-21

**Authors:** Jianfeng Duan, Minhua Cheng, Yali Xu, Yan Chen, Tao Gao, Qin Gu, Wenkui Yu

**Affiliations:** 1 Medical School of Nanjing University, 22nd Hankou Road, Nanjing, Jiangsu People’s Republic of China; 2 Department of Critical Care Medicine, The Affiliated Drum Tower Hospital, Nanjing University Medical School, 321st Zhongshan Road, Nanjing, Jiangsu People’s Republic of China

**Keywords:** Melatonin, Skeletal muscle wasting, Hypothalamic, Neuropeptides, Sepsis

## Abstract

To investigate whether exogenous melatonin (MLT) could alleviate skeletal muscle wasting by regulating hypothalamic neuropeptides expression. Adult male Sprague Dawley rats were intraperitoneally injected with lipopolysaccharide (LPS) (10 mg/kg), followed by MLT (30 mg/kg/day) or saline for 3 days. Hypothalamic tissues and skeletal muscle were obtained on day 3. Skeletal muscle wasting was measured by the mRNA expression of two E3 ubiquitin ligases, muscle atrophy F-box and muscle ring finger 1 as well as 3-methylhistidine (3-MH) and tyrosine release. Three hypothalamic neuropeptides (POMC, AgRP, CART) expression were detected in all groups. POMC expression knockdown was achieved by ARC injection of lentiviruses containing shRNA against POMC. Two weeks after ARC viruses injection, rats were i.p. injected with LPS (10 mg/kg) followed by MLT (30 mg/kg/day) or saline for 3 days. Brain tissues were harvested for immunostaining. In septic rats, 3-MH, tyrosine release and muscle atrophic gene expression were significantly decreased in MLT treated group. POMC and CART expression were lower while AgRP expression was higher in MLT treated group. Furthermore, in septic rats treated with MLT, muscle wasting in those with lower expression of neuropeptide POMC did not differ from those with normal POMC expression. Exogenous MLT could alleviate skeletal muscle wasting in septic rats by regulating hypothalamic neuropeptides.

## Introduction

Melatonin (MLT) is a pineal hormone that maintains normal circardian rhythm [1]. MLT and its metabolites modulate a variety of molecular signaling pathways including proliferation, apoptosis, metastasis, inflammation and so on [2–4]. Recent studies have shown that MLT may be useful in the treatment of sepsis and septic injury due to its antioxidative and anti-inflammatory actions [5]. Further research suggested that MLT blocked NF-κB signaling induced by LPS through inhibiting the nuclear translocation and DNA-binding activity of the NF-κB p50 subunit [6]. These results indicate a promising therapeutic application for MLT in the treatment of sepsis.

Sepsis is a life-threatening disease triggered by the invasion of microbes and dysregulation of innate immune system [7]. Hypercatabolism occurs in the early phase of sepsis and can cause major metabolism disorders, among which, high protein catabolism and muscle wasting are supposed to be the main contributor to morbidity and mortality [8, 9]. Our previous research suggested that central regulation, especially hypothalamic arcuate nucleus (ARC) played a pivotal role in muscle wasting of septic animal models [10]. ARC is composed of two populations of neurons, POMC and AgRP neurons [11]. The former expresses anorexigenic peptides, POMC and CART, resulting in negative energy balance. The later expresses orexigenic peptides, NPY and AgRP, resulting in positive energy balance [12]. In our study, we found that increased expression of POMC was associated with skeletal muscle wasting in septic rats and that suppression of POMC expression could significantly alleviate septic skeletal muscle wasting [13]. Thus, the regulation of the expression of certain hypothalamic neuropeptides might be a possible treatment target for alleviating muscle wasting and improving prognosis of sepsis.

As mentioned previously, MLT administration was benificial to septic animal models due to its potent anti-inflammatory and antioxidant properties, but whether MLT could affect septic skeletal muscle wasting and hypothalamic neuropeptides remains uncertain. In this study, we hypothesized that MLT could alleviate skeletal muscle wasting by regulating certain hypothalamic neuropeptides expression.

## Materials and Methods

### Animals

Adult male Sprague–Dawley rats (250 ± 20 g) were obtained from the Animal Research Center, Jinling Hospital, Nanjing, China. The animals were raised under regular lighting conditions (12 h:12 h light cycle) in a constant temperature environment with free access to tap water and standard rat pellet chow. The experimental protocols were approved by the Institutional Animal Care and Use Committee of Nanjing University and Jinling Hospital.

### Study Protocol

All rats were housed at least 7 days to adapt to the environment before any experiment. Then a set of rats were randomly divided into four groups (n = 6 in each group): the MLT (MLT) group, the control (CON) group, the sham (sham) group and sham + MLT (sham + MLT) group. All rats were i.p. injected with LPS (10 mg/kg, *Escherichia Coli* serotype 055: B5, Sigma, St.Louis, MO, USA) followed by MLT (30 mg/kg/day) for 3 days in the MLT group or saline (30 ml/kg/day) for 3 days in the CON group; and all rats were i.p. injected with saline(10 ml/kg) followed by MLT (30 mg/kg/day) for 3 days in the sham + MLT group or saline (30 ml/kg/day) for 3 days in the shan group. The selection of melatonin dosage 30 mg/kg/day was reference to many former publications, which is confirmed to be effective to reduced LPS-induced inflammation and metabolic alterations [14–17]. On day 3, the animals were sacrificed with an overdose of phenobarbital sodium. Extensor digitorum longus (EDL), gastrocnemius and hypothalamic tissue were obtained from each rat and kept at − 80 °C until analysis.

Knockdown of hypothamic POMC expression was realized by using a lentiviral method [13]. The lentiviral vector of shRNA against rat POMC and matched control was purchased from GenPharma (GenPharma Co., Ltd Shanghai). The sequences of shRNA was CUCUUCAAGAACGCCAUCA (5′–3′), whose interfering effect was confirmed in vitro. Lentiviruses were produced from HEK293T cells through cotransfection of target sequences with their packaging plasmids. Lentiviruses were purified by ultracentrifugation and ~ 1 × 10^9^ particles/site were used for each virus injection. The bilateral injections to the ARC were directed using an ultra-precise stereotax (Kopf Instruments) to the coordinates of 3.3 mm posterior to the bregma, 9.0 mm below the surface of the skull, and 0.3 mm lateral to midline. Purified lentivirus were injected over 10 min using a 5 μl Hamilton syringe attached to a microinfusion pump (World recision Instruments, Sarasot a, FL). The needle was left for an additional 5 min and then slowly withdrawn.

Another set of rats were first lateral ventricular catheterized. Then all rats were randomly categorized into four groups (n = 6 in each group): POMC knockdown and MLT treated group (PM group), POMC knockdown and saline treated group (PS group), normal POMC expression and MLT treated group (VM group), normal POMC expression and saline treated group (VS group). The PM and PS groups were injected with interfering virus to the ARC, the VM and VS groups was injected with control virus. Two weeks later, all animals were i.p. injected with LPS (10 mg/kg) followed by MLT (30 mg/kg/day) for 3 days in both PM and VM groups and saline (30 ml/kg/day) for 3 days in the PS and VS group. Then all animals were sacrificed with an overdose of phenobarbital sodium. EDL, gastrocnemius and hypothalamic tissue were obtained from each rat and kept at − 80 °C until analysis.

### Measurement of Protein Breakdown Rates in EDL

High performance liquid chromatography(HPLC) was used to measure protein breakdown rates, as formerly described, fresh EDL muscles were fixed via the tendons to aluminium wire supports at resting length, and preincubated in oxygenated medium (95% O_2_–5% CO_2_); Krebs–Henseleit bicarbonate buffer (pH7-4) which contains 5 mM glucose, 0–1 U/ml insulin, 0–1 mM isoleucine, 0–17 mM leucine and 0–20 mM valine. After 1 h preincubation, muscles were transferred to fresh medium of identical composition and incubated for a further 2 h with 0–5 mM cycloheximide. The degradation rates of total and myofibrillar proteins were measured by the release in the medium of free tyrosine and 3-methyl-histidine (3-MH) respectively, and expressed as nanomoles of tyrosine/3-MH in medium per 2 h/g/muscle. Muscle was also homogenized in 0–4 mM perchloric acid to determine tissue-free 3-MH and tyrosine. The net generation of 3-MH was calculated as the amount of 3-MH in the medium minus the decrease in tissue free 3-MH before and after incubation. Net free tyrosine generation was calculated as the amount of tyrosine released into the medium plus the increase in tissue-free tyrosine during incubation. Both tyrosine and 3-MH levels in medium or tissue samples were measured by high-performance liquid chromatography (HPLC).

### Measuremnt of Muscle Atrophic Gene, Hypothalamic Neuropeptides

Real-time PCR was used to detect gene expression. The total RNA was isolated from hypothalamus and gastrocnemius muscle using Trizol reagent (Invitrogen, USA) according to the manufacturer’s instructions. Gene expression was analysed using the Rotor-Gene Real-Time Analysis Software 6.1.Glyceraldehyde phosphate dehydrogenase (GAPDH) was used as an internal control gene to normalize the target mRNAs, and gene expression was compared among groups using the DDCT method. The primer sequences are listed in Table 1.

**Table 1:** Primers for RT-PCR assay

Gene		Primers
MuRF-1	Forward	5′-GGACGGAAATGCTATGGAGA-3′
	Reverse	5′-AACGACCTCCAGACATGGAC-3′
MAFbx	Forward	5′-CCATCAGGAGAAGTGGATCTATGTT-3′
	Reverse	5′-ATGACGTG AAACCCCCTTCG-3′
POMC	Forward	5′-CCTCCTGCTTCAGACCTCCA-3′
	Reverse	5′-GGCTGTTCATCTCCGTTGC-3′
AgRP	Forward	5′-TGAAGGGCATCAGAAGGT-3′
	Reverse	5′-CACAGGTCGCAGCAAGGT-3′
CART	Forward	5′-CCGAGCCCTGGACATCTA-3′
	Reverse	5′-GGAATGCGTTTACTCTTGAGC-3′
GAPDH	Forward	5′-GCAAGTTCAACGGCACAG-3′
	Reverse	5′-GCCAGTAGACTCCACGACAT-3′

### Western Blotting

Animal tissues were homogenized and incubated for 60 min at 4 °C in lysis buffer and separated by SDS/PAGE for Western blot analyses. Primary antibodies included POMC, CART, AgRP andβ-actin (Cell Signaling Technology, Inc.). Secondary antibody was HRP-conjugated anti-rabbit IgGs (Pierce). The densitometric analyses of Western blotting images were performed using Image-Pro Plus software (Media Cybernetics).

### Immunofluorescence Analysis

Rats were anaesthetized with isoflurane (4% induction and 1.5–2% maintenance) and transcardially perfused with 200 ml of saline containing heparin (50 i.u./l), followed by 400 ml of 4% paraformaldehyde in 0.1 M phosphate-buffered saline (pH 7.2). The brains were dissected out and post-fixed by immersion for 1 h at RT in the same fixative. Later, brains were cryoprotected with a 30% sucrose solution in 0.1 M PB at – 20 °C and brain sections of 6 μm thickness were obtained using a freezing-sliding microtome. Sections were washed in a PBS solution (0.1 M, pH 7.4) and then stored at 4 °C in a freezing solution (30% glycerol and 30% ethylene glycol in 0.1 M PB at pH 7.4). Fixed brain sections were rinsed in PBS with 0.2% TritonX-100, and then blocked for 2 h with 1% BSA and 5% normal serum in PBS-Tx. After incubated overnight at 4 °C with 1% BSA, 2% normal serum and primary rabbit anti-POMC antibodies, and subsequently reacted with FITC-labeled Goat Anti-Rabbit secondary antibody (Invitrogen). The nucleus was stained with DAPI (4,6-diamidino-2-phenylindole). Images were captured using a FW1000 confocal microscope.

### Hematoxylin–Eosin Staining of EDL

EDL specimens obtained during the rat experiment were immediately fixed in 10% paraformaldehyde and incubated overnight at room temperature. Next, tissue samples were embedded in paraffin and 5-µm sections were cut. Sections were deparaffinized in xylene and rehydrated in graded ethanol to distilled water and stained with hematoxylin and eosin for histological analysis. Morphological changes were observed using light microscopy, by an independent pathologist.

### Statistical Analysis

The experimental data were expressed as means ± standard error (SE). Statistical analyses were performed using SPSS for Windows version 23.0 (SPSS Inc., Chicago, IL). The comparisons of differences among groups were accomplished by a two-way analysis of variance (ANOVA), with treatment (MLT and saline) as the main factor, followed by Newman–Keuls post hoc test. Differences were considered statistically significant at P < 0.05.

## Results

### Effect of Exogenous Melatonin on Skeletal Muscle Wasting and Hypothalamic Neuropeptides Expression in Septic Rats

#### EDL Weight and Body Weight Change

**Fig. 1:**
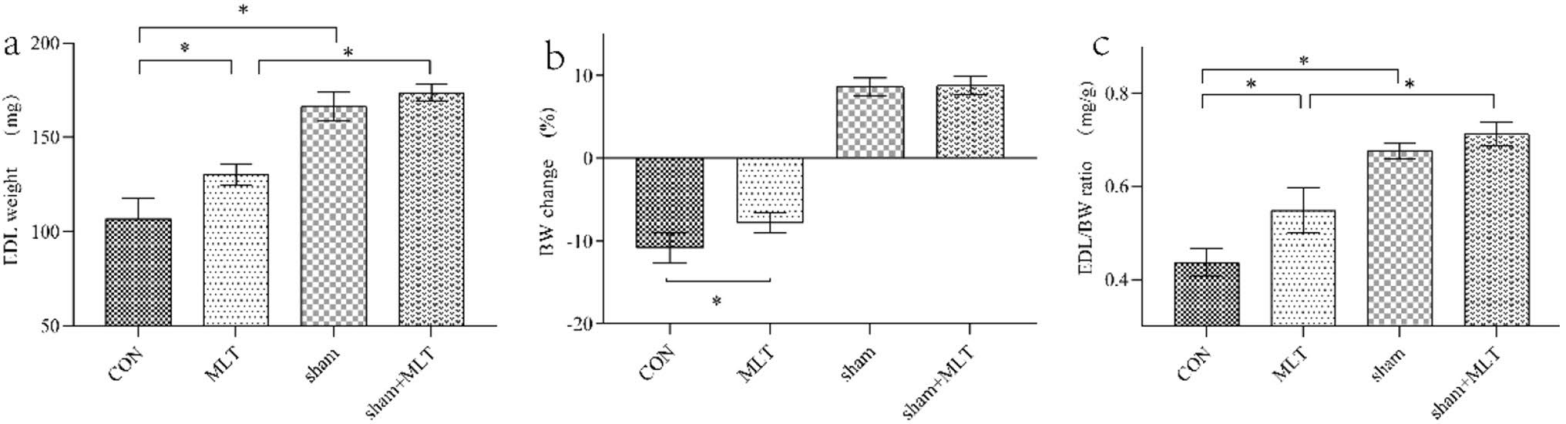
EDL weight and body weight change. **a** EDL weight, **b** BW change, **c** EDL/BW ratio. A significant difference was labeled (*) with P values < 0.05. *EDL* extensor digitorum longus, *BW* body weight

Body weight (BW) was recorded at day 0 and day 3. EDL weight was measured immediately after the muscle was separated. EDL weight from the MLT group was significantly heavier than that from the CON group (*P* < 0.01, Fig. 1) and body weight decrease was much lower in the MLT group than the CON group (*P* < 0.05, Fig. 1). Also, EDL/BW ratio was significantly higher in MLT group than in CON group (*P* < 0.01, Fig. 1). However, there was no significant difference between sham group and sham + MLT group in EDL weight or body weight change (*P* > 0.05, Fig. 1).

#### Rate of Skeletal Muscle Protein Breakdown and Muscle Atrophic Gene Expression

**Fig. 2:**
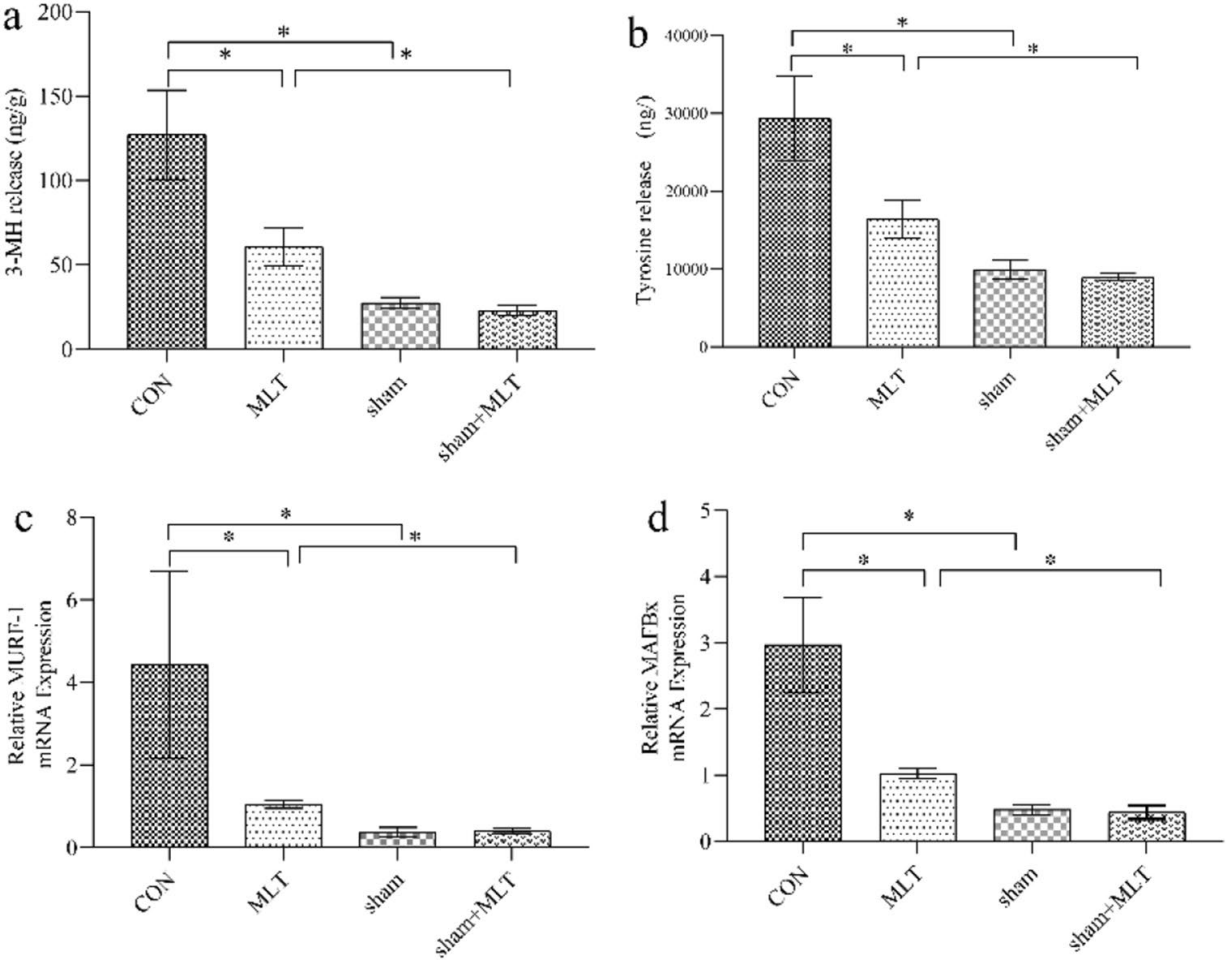
Rate of skeletal muscle protein breakdown and muscle atrophic gene expression **a**, **b** 3-MH and tyrosine release in EDL were measured by high performance liquid chromatography(HPLC); **c**, **d** muscle atrophic gene expression in gastrocnemius were measured by Real-time PCR. A significant difference was labeled (*) with P values < 0.05

Skeletal muscle protein breakdown was measured by 3-MH and tyrosine release. As expected, in LPS rats, when compared with CON group, there was a significant decrease in the rate of total protein proteolysis after MLT administration (both *P* < 0.01, Fig. 2). Significant reduced expression of two atrophic gene, MuRF-1 and MAFbx was observed in MLT group (*P* < 0.05 and *P* < 0.01, Fig. 2). However, MLT had no detectable effect on skeletal muscle metabolism in saline-injected rats. These results demonstrated that MLT could alleviate skeletal muscle wasting in septic rats.

#### Hematoxylin–Eosin Staining of EDL

**Fig. 3:**
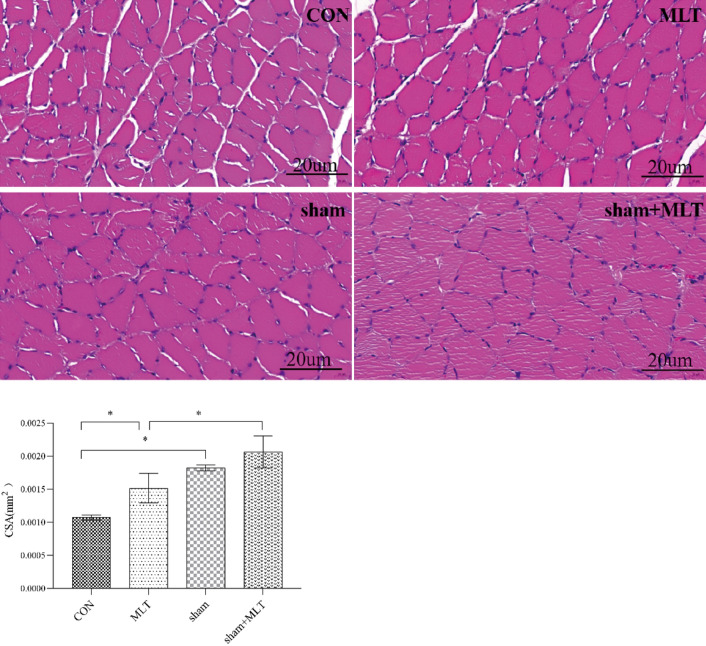
HE staining of EDL. A significant difference was labeled (*) with P values < 0.05. CSA, cross sectional area

HE staining of EDL was performed to further demonstrate the effect of MLT on skeletal muscle degradation in septic rats. As shown, muscle fiber in MLT group was denser than that in CON group, but there was no significant difference between sham group and sham + MLT group in muscle fiber (Fig. 3).

#### Hypothalamic Neuropeptides Expression

**Fig. 4:**
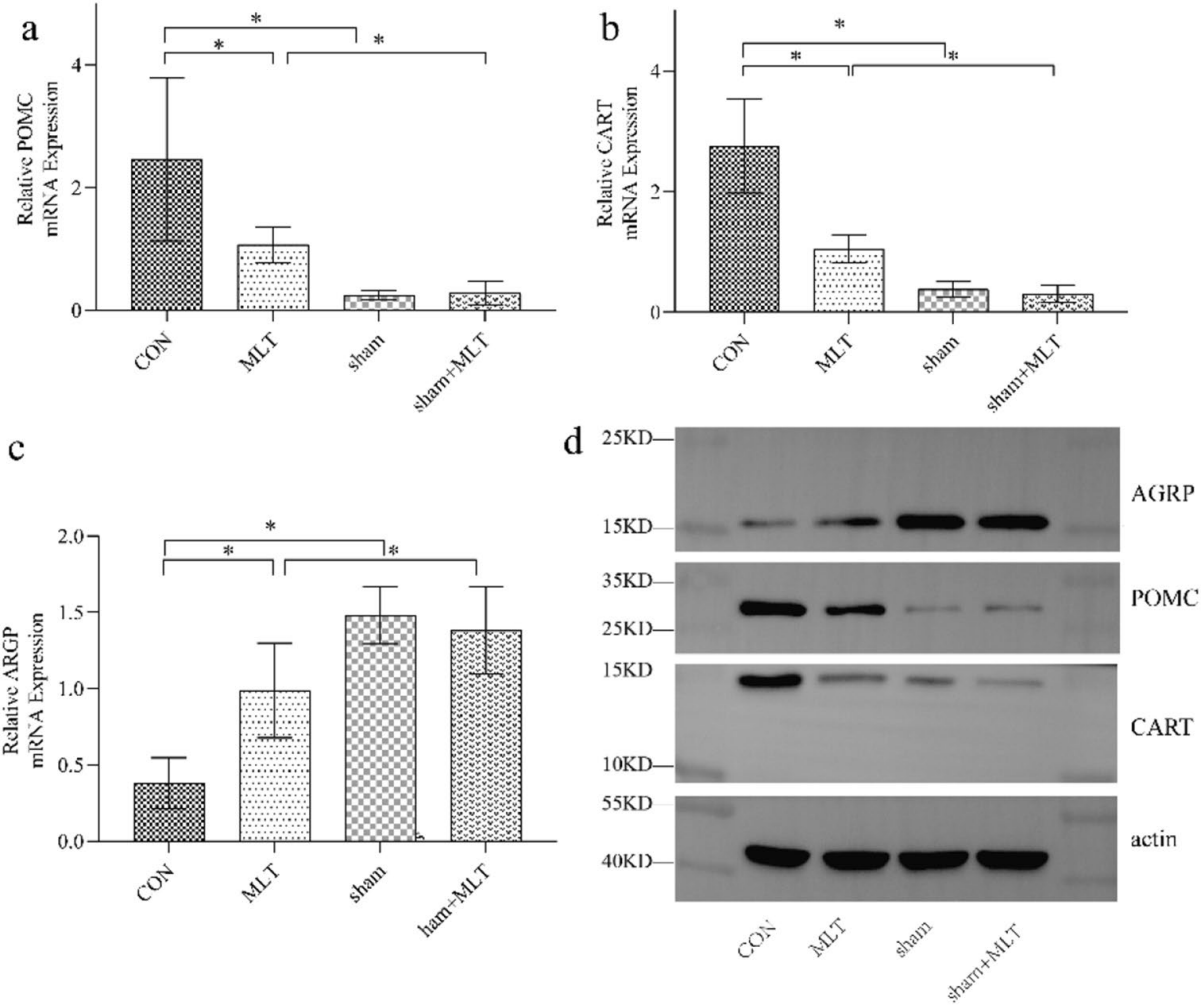
Hypothalamic neuropeptides expression, **a** POMC, **b** CART, **c** AGRP expression tested with RT-PCR; A significant difference was labeled (*) with P values < 0.05, **d** hypothalamic neuropeptides expression measured with Western Blot

In both RT-PCR and western blotting analysis, the anorexigenic genes, POMC and CART, expression decreased after MLT administration when compared with the CON group (*P* < 0.01 and *P* < 0.05, Fig. 4). On the contrary, there was a significant increase in orexigenic neuropeptide AgRP expression in the control group (P < 0.01, Fig. 4).

#### Analysis of the Correlation Between Skeketal Muscle Wasting and Hypothalamic Neuropeptides Expression

**Fig. 5:**
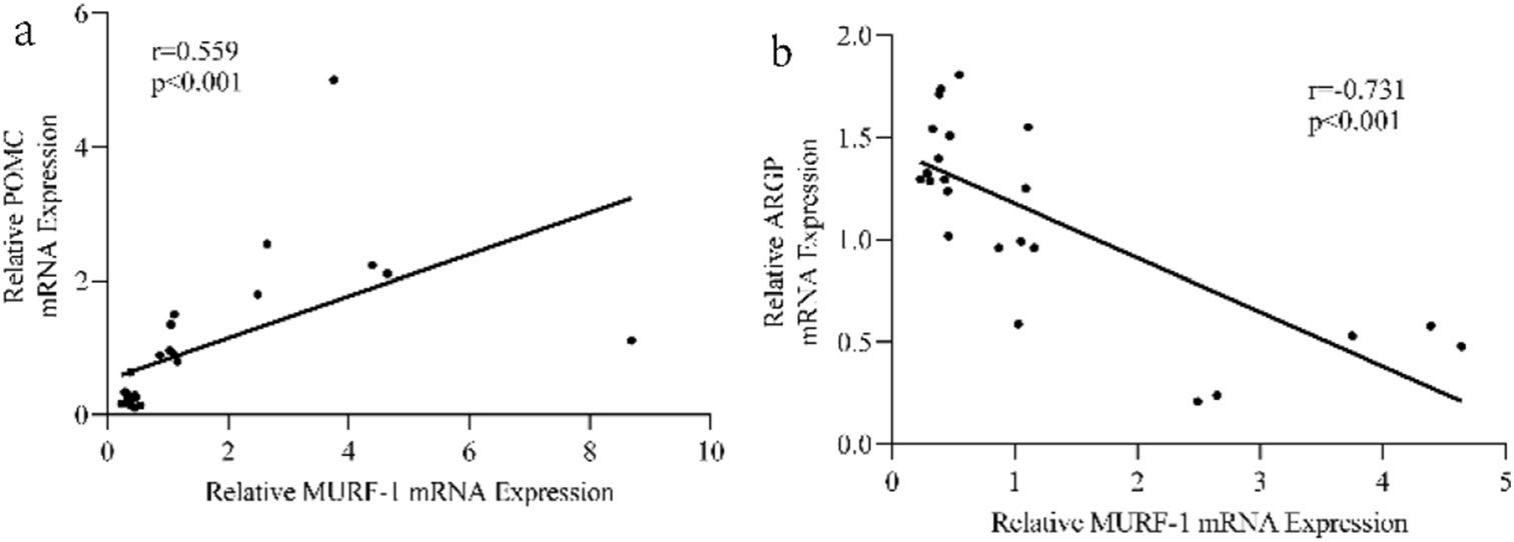
Analysis of the correlation between skeketal muscle wasting and hypothalamic neuropeptides expression. **a** There was a significant positive correlation between hypothalamic POMC expression and MuRF-1 expression (r = 0.559, P < 0.05). **b** A negative correlation was found between hypothalamic AgRP expression and MuRF-1 expression (r = − 0.731, P < 0.05)

For further illustration of the relationship between skeletal muscle wasting and hypothalamic neuropetides, a correlation analysis was performed. We chose MuRF-1 expression to represent muscle degradation due to that MAFbX gene is also involved in the process of muscle synthesis. There was a significant correlation between hypothalamic POMC expression and MuRF-1 expression (r = 0.559, *P* < 0.05, Fig. 5). Also, a negative correlation was found between hypothalamic AgRP expression and MuRF-1 expression (r = -0.731 *P* < 0.05, Fig. 5).

### Effect of Hypothamic POMC Expression Knockdown on Skeletal Muscle Wasting in Septic Rats

#### Hypothalamic POMC Expression

**Fig. 6:**
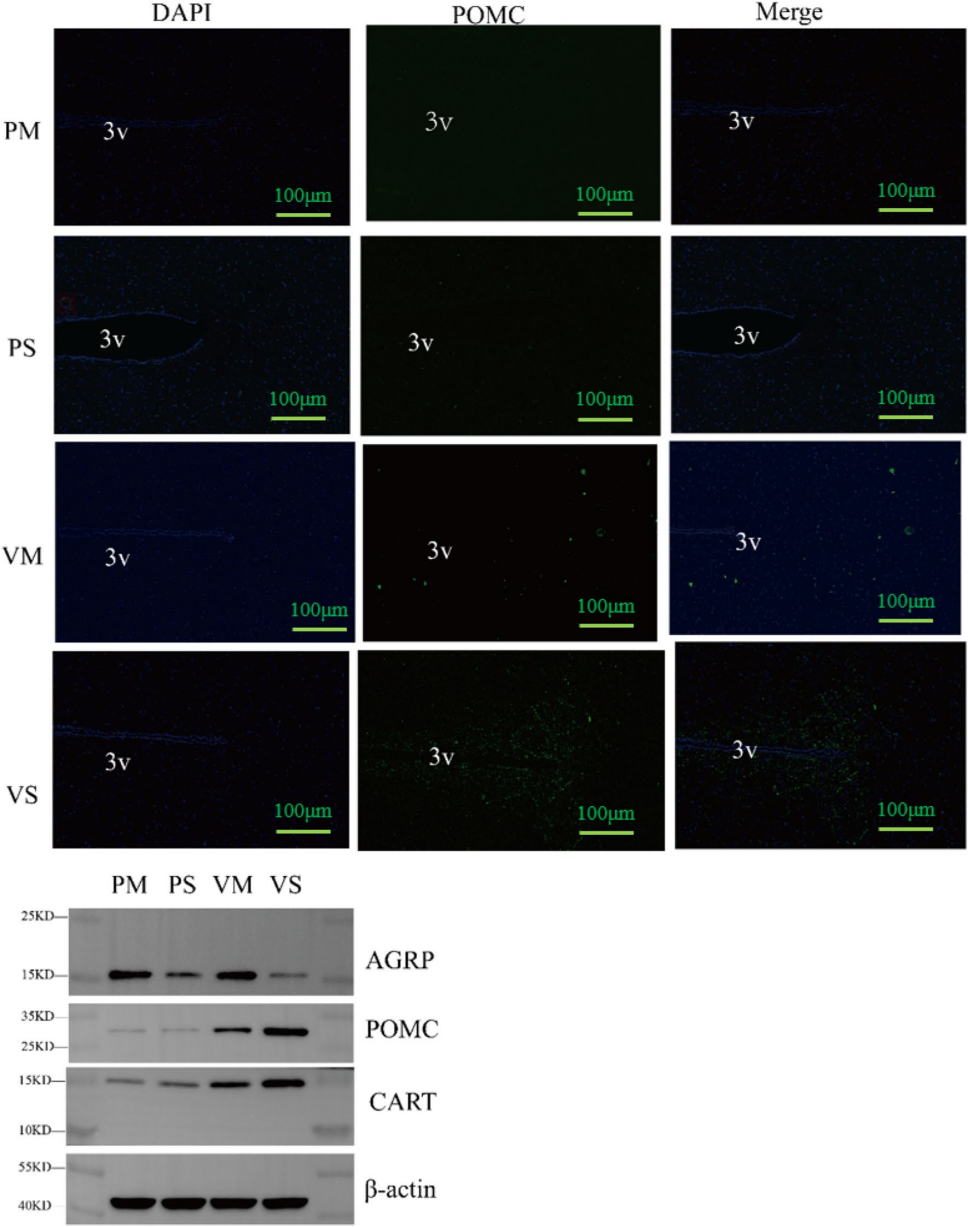
Hypothalamic neuropeptide expression in immunostaining and western blotting in saline or MLT treated rats after ARC injection of lentiviruses containing shRNA against POMC. POMC immunostaining (green) across the hypothalamic ARC of rats in experiment. DAPI staining (blue) reveals the nucleus of all cells in the sections. PM, POMC knockdown and MLT treated group; PS, POMC knockdown and saline treated group; VM, normal POMC expression and MLT treated group. VS, normal POMC expression and saline treated group

Site-specific RNA interference via a hypothalamic delivery of lentiviral shRNA against rat POMC was used to knock down POMC expression. By applying POMC immunostaining and mRNA test, it was confirmed that site specific POMC knockdown was successful. As a result, in both immunostaining and western blotting analysis, POMC expression was significantly less in PM and PS group (Fig. 6).

#### EDL Weight and Body Weight Change

**Fig. 7:**
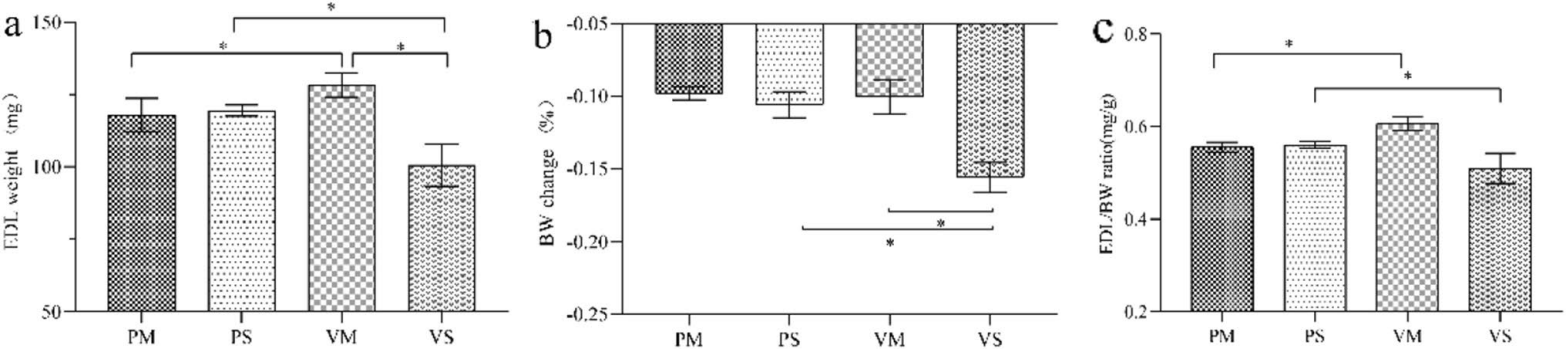
EDL weight and body weight change in saline or MLT treated rats after ARC injection of lentiviruses containing shRNA against POMC. **a** EDL weight, **b** BW change, **c** EDL/BW ratio. A significant difference was labeled (*) with P values < 0.05

Body weight (BW) was recorded at day 0 and day 3. EDL weight was measured immediately after the muscle was separated. EDL weight and EDL/BW ratio were similar in both POMC knockdown groups with or without MLT treatment. (*P* > 0.05, Fig. 7). While in MLT-treated rats, both EDL weight and EDL/BW ratio were significantly lower in the POMC knockdown group than the vehicle-treated group (*P* < 0.05, Fig. 7).

#### Hematoxylin–Eosin Staining of EDL

**Fig. 8:**
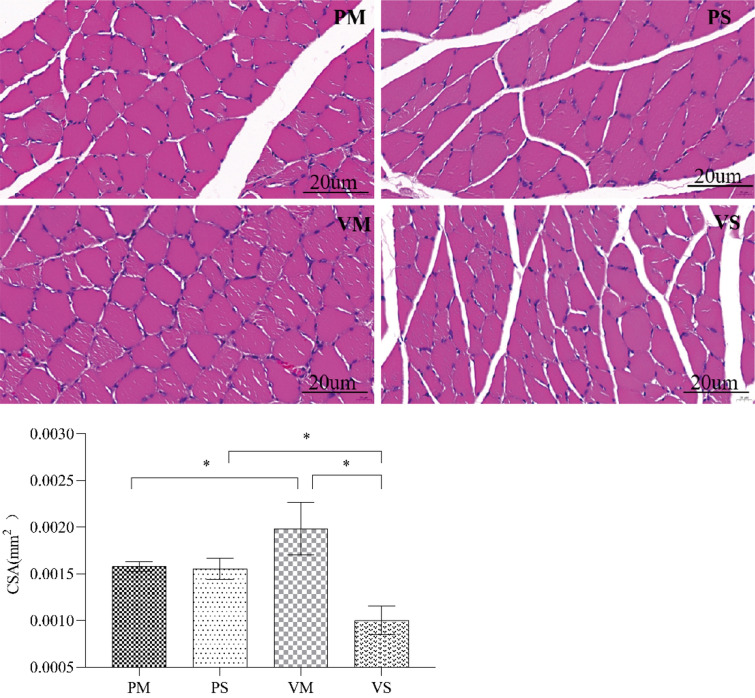
HE staining of EDL in saline or MLT treated rats after ARC injection of lentiviruses containing shRNA against POMC. A significant difference was labeled (*) with P values < 0.05

Muscle fiber was denser in the VM group than PM group, and there was no significant difference between PM group and PS group (Fig. 8).

#### Rate of Skeletal Muscle Protein Breakdown and Muscle Atrophic Gene Expression

**Fig. 9:**
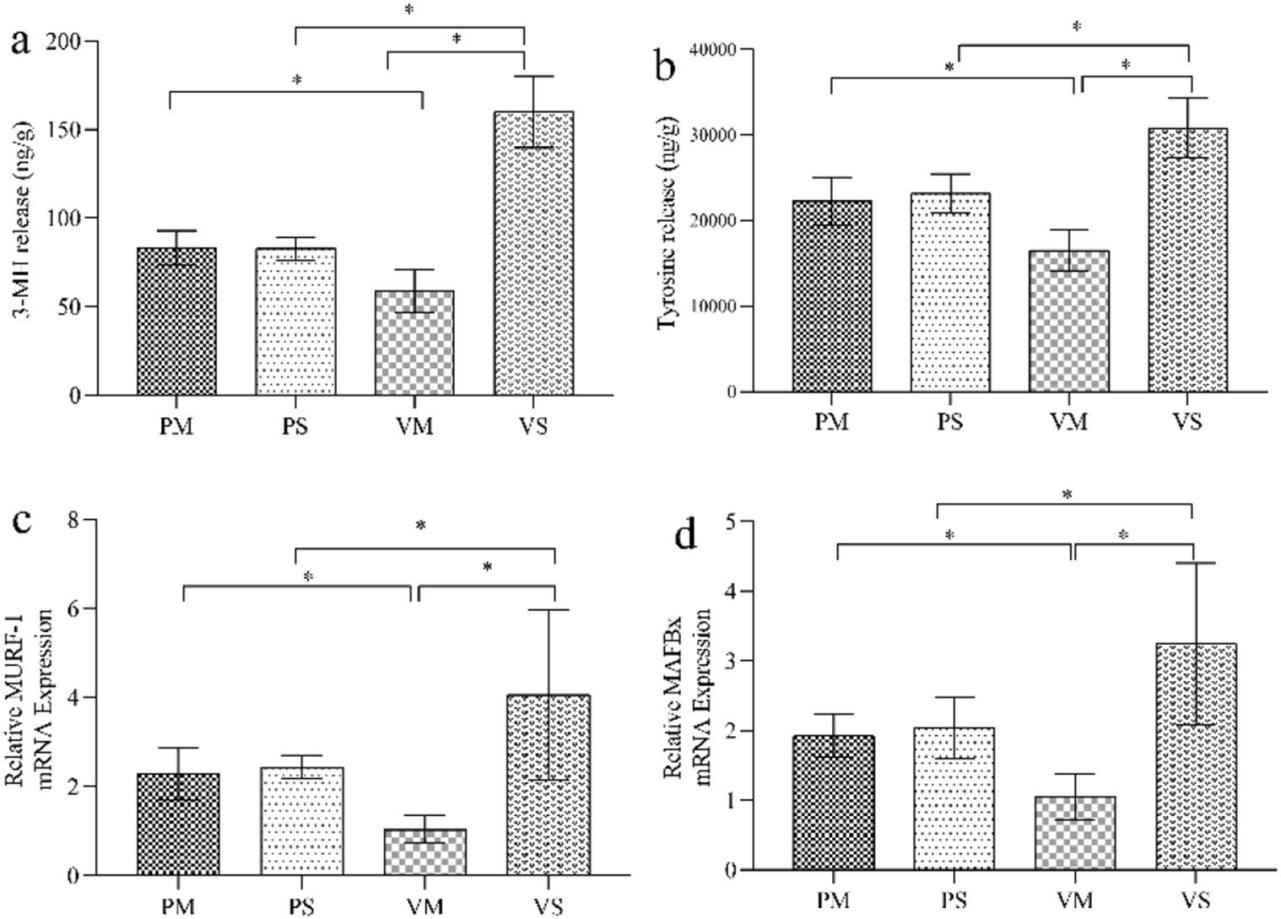
Rate of skeletal muscle protein breakdown and muscle atrophic gene expression in saline or MLT treated rats after ARC injection of lentiviruses containing shRNA against POMC. **a**, **b** 3-MH and tyrosine release in EDL were measured by high performance liquid chromatography(HPLC); **c**, **d** muscle atrophic gene expression in gastrocnemius were measured by Real-time PCR. A significant difference was labeled (*) with P values < 0.05

There were no differences in 3-MH, tyrosine release or mRNA expression of MuRF-1 and MAFbx between the two POMC knockdown groups with or without MLT treatment (both *P* > 0.05, Fig. 9). Instead, in the MLT-treated rats, increased 3-MH and tyrosine release were observed in the POMC knockdown group than in vehicle-treated group. Similarly, the mRNA expression of MuRF-1 and MAFbx were significantly higher in POMC knockdown group (both *P* < 0.05, Fig. 9). Taken together, these results demonstrated that MLT alleviating septic muscle wasting might be associated with POMC expression.

## Discussion

In this study, we first demonstrated that MLT could alleviate muscle wasting and regulate certain hypothalamic neuropeptides expression in sepsis animal models. Then by knockdown a key hypothalamic neuropeptide, POMC, we found that MLT’s capacity of alleviating muscle wasting was weakened. Taken together, these results indicated that MLT could alleviate muscle wasting by regulating the expression of POMC.

As potent antioxidants and free radical scavengers, MLT and its metabolites are protective against a variety of disorders [18–20]. MLT significantly improved median survival times and survival rates after a lethal dose of LPS [21]. MLT reduces pro-inflammatory markers by inhibiting the expression of TNF-α, IL-1β and IL-6, thus limits the severity of inflammatory diseases induced by oral bacteria or LPS [22–24]. In human trials, it has been shown that oral supplementation of melatonin, before strenuous exercise, was able to reduce plasma pro-inflammatory TNF-α and IL-6, and to increase the anti-inflammatory IL-1β cytokine [25]. In a number of animal models of septic shock, as well as in patients with septic disease, MLT exerts beneficial effects on cellular damage and multiorgan failure by acting through a variety of mechanisms, like immunomodulation or direct or indirect antioxidant activity [26].

Sepsis is defined as life-threatening organ dysfunction caused by a dysregulated host response to infection [7]. Skeletal muscle tissue comprises 50–60% of body cell mass and represents the largest organ affected by exaggerated whole-body inflammatory response caused by sepsis [27]. As a serious complication of sepsis, skeletal muscle energy metabolism is rapidly altered during sepsis. Skeletal muscle wasting signifies hypercatabolism and predicts worse prognosis [9]. Muscle wasting contributes to prolonged mechanical ventilation and ICU stay, as well as severe infection and mortality [28, 29]. Studies in animal models and patients with sepsis have provided evidence that myofibrillar proteins are particularly sensitive to the effects of sepsis [30]. Ozkok E et al. investigated the effects of MLT on tissue structure, energy metabolism in skeletal muscle, and antioxidant level in rats with endotoxemia. Results showed that MLT treatment prevented muscle damage by increasing ATP and glutathione levels [14]. Moreover, in our previous researches, we demonstrated that hypothalamic inflammation could result in skeletal muscle wasting in septic rats [10]. MLT’s anti-inflammatory effect, as to reduce hypothalamic inflammation in the present study, might be one of the mechanisms for alleviating septic skeletal muscle wasting. When respiratory muscles are affected in sepsis or critically ill patients, it could lead to prolonged mechanical ventilation and severe pulmonary infection. We speculate that the positive effects of MLT on respiratory muscles may also account for less multiple organ dysfunction and better prognosis in critically ill induced by MLT.

We also demonstrated that exogenous MLT could affect certain hypothalamic neuropeptides expression. Recently, Bo Gao et al. reported that MLT can attenuate the IL-1β-induced activation of the NF-κB signaling pathway [31]. As in our previous research, it is revealed that hypothalamic inflammatory response induced by NF-κB/IL-1β pathway is assoicated with the expression of certain neuropeptides in septic rats [13]. Thus, hypothalamic NF-κB/IL-1β inflammation pathway might account for MLT’s positive effect on neuropeptides expression. On the other hand, MLT may regulate hypothalamic neuropeptides through the AMPK signaling pathway. Some studies demonstrated that there exists a positive feedback regulatory mechanism between the activation of AMPK-α1 and ROS, and the anti-oxidant agent MLT would possibly normalize the APMK level in brain [32–34]. In our previous we found that hypothalamic AMPK-induced autophagy ameliorates hypercatabolism in septic rats by regulating POMC expression [13]. Therefore, AMPK-related pathways may also be one of the mechanisms by which melatonin affects the expression of neuropeptide in the hypothalamus. Hypothalamic neuropeptides expression levels, especially POMC and AgRP were suggested to be associated with skeletal muscle wasting [11]. Besides the fact that MLT has the potency to get through blood brain barrier, in septic animal models, endothelial injury and increased capillary permeability would result in increased permeability of blood brain barrier [35]. Thus, exogenous MLT could work directly on hypothalamus. Taken together, MLT alleviated skeletal muscle wasting by regulating certain hypothalamic neuropeptides expression, and hypothalamic inflammation pathway might play an important role in the process, which needs further confirmation.

To further explore the exact mechanism of MLT alleviating skeletal muscle wasting, we inhibited the expression of a key neuropeptide, POMC, after LPS administration by using a lentiviral method. Erenow, we also successfully inhibited the elevated expression of POMC after LPS treatment using lentiviral method, and found that knockdown of POMC effectively ameliorated peripheral muscle wasting induced by LPS but hypothalamic NF-κB pathway, inflammation and other hypothalamic neuropeptides caused by LPS were unaffected. It indicates that NF-κ B pathway is more likely to be the upstream of POMC and the peripheral effect on muscle wasting was more likely related to POMC alone. Remarkably, in the two knockdown groups of present study, skeletal muscle wasting was similar with or without MLT treatment. However, in the animals treated with MLT, skeletal muscle wasting was significantly mitigated in the vehicle-treated group when compared with the knockdown group. This result furtherly indicated that POMC expression was essential in the process of exogenous MLT alleviating LPS-induced skeletal muscle wasting.

Our study was the first to demonstrate that MLT could alleviate skeletal muscle wasting by regulating hypothalamic POMC expression in septic animals models. It is well known that hypothalamus is the centre of energy metabolism. In recent decades, emerging data have suggested that central regulation may also contributed to the metabolic and behavioral actions in critical illness or cachexic conditions [36]. Inhibition of hypothalamic TNF signaling can partially restore body weight, increase food intake and enhanced survival rate in septic animal models [37]. Here our study further confirmed the role of hypothalamic regulation in septic muscle wasting. Even though the present study was the first to demonstrate the fact, which MLT could alleviate septic skeletal muscle wasting by regulating hypothalamic POMC expression, there are some limitations. Firstly, we only used shRNA method to inhibit neuropeptides expression, which was inferior to the gene knockout approach, especially the cre-loxp method. Secondly, we only chose 72 h to test muscle wasting due to LPS-injection method, longer time span using other model are warranted to confirm the results. Furtherly, the exact molecular mechanism underlying how MLT act on POMC and how POMC regulate septic skeletal muscle wasting required more research.

## Data Availability

The datasets used and/or analysed during the current study are available from the corresponding author on reasonable request.

## References

[1] Reiter RJ (1991). Pineal melatonin: cell biology of its synthesis and of its physiological interactions. Endocr Rev.

[2] Galano A, Tan DX, Reiter RJ (2011). Melatonin as a natural ally against oxidative stress: a physicochemical examination. J Pineal Res.

[3] Galano A, Tan DX, Reiter RJ (2013). On the free radical scavenging activities of melatonin's metabolites, AFMK and AMK. J Pineal Res.

[4] Radogna F, Diederich M, Ghibelli L (2010). Melatonin: a pleiotropic molecule regulating inflammation. Biochem Pharmacol.

[5] Srinivasan V, Pandi-Perumal SR, Spence DW, Kato H, Cardinali DP (2010). Melatonin in septic shock: some recent concepts. J Crit Care.

[6] Choi EY, Jin JY, Lee JY, Choi JI, Choi IS, Kim SJ (2011). Melatonin inhibits Prevotella intermedia lipopolysaccharide-induced production of nitric oxide and interleukin-6 in murine macrophages by suppressing NF-kappaB and STAT1 activity. J Pineal Res.

[7] Singer M, Deutschman CS, Seymour CW, Shankar-Hari M, Annane D, Bauer M, Bellomo R, Bernard GR, Chiche JD, Coopersmith CM (2016). The Third International Consensus Definitions for Sepsis and Septic Shock (Sepsis-3). JAMA.

[8] Puthucheary ZA, Rawal J, McPhail M, Connolly B, Ratnayake G, Chan P, Hopkinson NS, Phadke R, Dew T, Sidhu PS (2013). Acute skeletal muscle wasting in critical illness. JAMA.

[9] Angus DC, Linde-Zwirble WT, Lidicker J, Clermont G, Carcillo J, Pinsky MR (2001). Epidemiology of severe sepsis in the United States: analysis of incidence, outcome, and associated costs of care. Crit Care Med.

[10] Duan K, Yu W, Lin Z, Tan S, Bai X, Gao T, Xi F, Li N (2014). Endotoxemia-induced muscle wasting is associated with the change of hypothalamic neuropeptides in rats. Neuropeptides.

[11] Arora S (2006). Anubhuti: role of neuropeptides in appetite regulation and obesity—a review. Neuropeptides.

[12] Oyama LM, do Nascimento CM, Carnier J, de Piano A, Tock L, de LimaSanches P, Gomes FA, Tufik S, de Mello MT, Damaso AR (2010). The role of anorexigenic and orexigenic neuropeptides and peripheral signals on quartiles of weight loss in obese adolescents. Neuropeptides.

[13] Duan K, Chen Q, Cheng M, Zhao C, Lin Z, Tan S, Xi F, Gao T, Shi J, Shen J (2016). Hypothalamic activation is essential for endotoxemia-induced acute muscle wasting. Sci Rep.

[14] Ozkok E, Yorulmaz H, Ates G, Aksu A, Balkis N, Sahin O, Tamer S (2016). Amelioration of energy metabolism by melatonin in skeletal muscle of rats with LPS induced endotoxemia. Physiol Res.

[15] An R, Zhao L, Xi C, Li H, Shen G, Liu H, Zhang S, Sun L (2016). Melatonin attenuates sepsis-induced cardiac dysfunction via a PI3K/Akt-dependent mechanism. Basic Res Cardiol.

[16] Chen J, Xia H, Zhang L, Zhang H, Wang D, Tao X (2019). Protective effects of melatonin on sepsis-induced liver injury and dysregulation of gluconeogenesis in rats through activating SIRT1/STAT3 pathway. Biomed Pharmacother.

[17] Fink T, Glas M, Wolf A, Kleber A, Reus E, Wolff M, Kiefer D, Wolf B, Rensing H, Volk T (2014). Melatonin receptors mediate improvements of survival in a model of polymicrobial sepsis. Crit Care Med.

[18] Cuzzocrea S, Reiter RJ (2002). Pharmacological actions of melatonin in acute and chronic inflammation. Curr Top Med Chem.

[19] Cuzzocrea S, Reiter RJ (2001). Pharmacological action of melatonin in shock, inflammation and ischemia/reperfusion injury. Eur J Pharmacol.

[20] Galano A, Reiter RJ (2018). Melatonin and its metabolites vs oxidative stress: from individual actions to collective protection. J Pineal Res.

[21] Crespo E, Macias M, Pozo D, Escames G, Martin M, Vives F, Guerrero JM, Acuna-Castroviejo D (1999). Melatonin inhibits expression of the inducible NO synthase II in liver and lung and prevents endotoxemia in lipopolysaccharide-induced multiple organ dysfunction syndrome in rats. FASEB J.

[22] Mishra A, Paul S, Swarnakar S (2011). Downregulation of matrix metalloproteinase-9 by melatonin during prevention of alcohol-induced liver injury in mice. Biochimie.

[23] Cuesta S, Kireev R, Garcia C, Forman K, Escames G, Vara E, Tresguerres JA (2011). Beneficial effect of melatonin treatment on inflammation, apoptosis and oxidative stress on pancreas of a senescence accelerated mice model. Mech Ageing Dev.

[24] Kireev RA, Tresguerres AC, Garcia C, Ariznavarreta C, Vara E, Tresguerres JA (2008). Melatonin is able to prevent the liver of old castrated female rats from oxidative and pro-inflammatory damage. J Pineal Res.

[25] Ochoa JJ, Diaz-Castro J, Kajarabille N, Garcia C, Guisado IM, De Teresa C, Guisado R (2011). Melatonin supplementation ameliorates oxidative stress and inflammatory signaling induced by strenuous exercise in adult human males. J Pineal Res.

[26] Srinivasan V, Spence DW, Moscovitch A, Pandi-Perumal SR, Trakht I, Brown GM, Cardinali DP (2010). Malaria: therapeutic implications of melatonin. J Pineal Res.

[27] Holecek M (2012). Muscle wasting in animal models of severe illness. Int J Exp Pathol.

[28] Hasselgren PO, Fischer JE (1998). Sepsis: stimulation of energy-dependent protein breakdown resulting in protein loss in skeletal muscle. World J Surg.

[29] Hasselgren PO, Menconi MJ, Fareed MU, Yang H, Wei W, Evenson A (2005). Novel aspects on the regulation of muscle wasting in sepsis. Int J Biochem Cell Biol.

[30] Wu LL, Tang C, Liu MS (2001). Altered phosphorylation and calcium sensitivity of cardiac myofibrillar proteins during sepsis. Am J Physiol Regul Integr Comp Physiol.

[31] Gao B, Gao W, Wu Z, Zhou T, Qiu X, Wang X, Lian C, Peng Y, Liang A, Qiu J (2018). Melatonin rescued interleukin 1beta-impaired chondrogenesis of human mesenchymal stem cells. Stem Cell Res Ther.

[32] Ju TC, Chen HM, Chen YC, Chang CP, Chang C, Chern Y (2014). AMPK-alpha1 functions downstream of oxidative stress to mediate neuronal atrophy in Huntington's disease. Biochim Biophys Acta.

[33] Kim JE, Kim YW, Lee IK, Kim JY, Kang YJ, Park SY (2008). AMP-activated protein kinase activation by 5-aminoimidazole-4-carboxamide-1-beta-D-ribofuranoside (AICAR) inhibits palmitate-induced endothelial cell apoptosis through reactive oxygen species suppression. J Pharmacol Sci.

[34] Rehman SU, Ikram M, Ullah N, Alam SI, Park HY, Badshah H, Choe K, Kim MO (2019). Neurological enhancement effects of melatonin against brain injury-induced oxidative stress, neuroinflammation, and neurodegeneration via AMPK/CREB signaling. Cells.

[35] Su SC, Reiter RJ, Hsiao HY, Chung WH, Yang SF (2018). Functional interaction between melatonin signaling and noncoding RNAs. Trends Endocrinol Metab.

[36] Krasnow SM, Marks DL (2010). Neuropeptides in the pathophysiology and treatment of cachexia. Curr Opin Support Palliat Care.

[37] Arruda AP, Milanski M, Romanatto T, Solon C, Coope A, Alberici LC, Festuccia WT, Hirabara SM, Ropelle E, Curi R (2010). Hypothalamic actions of tumor necrosis factor alpha provide the thermogenic core for the wastage syndrome in cachexia. Endocrinology.

